# *Lhb*^−/−^*Lhr*^−/−^ Double Mutant Mice Phenocopy *Lhb*^−/−^ or *Lhr*^−/−^ Single Mutants and Display Defects in Leydig Cells and Steroidogenesis

**DOI:** 10.3390/ijms232415725

**Published:** 2022-12-11

**Authors:** Zhenghui Liu, Mark Larsen, Zhenmin Lei, C. V. Rao, T. Rajendra Kumar

**Affiliations:** 1 Division of Reproductive Sciences, Department of Obstetrics and Gynecology, University of Colorado Anschutz Medical Campus, Aurora, CO 80045, USA; 2 Department of Obstetrics and Gynecology, University of Louisville School of Medicine, Louisville, KY 40202, USA; 3 Department of Cell Biology, Molecular and Human Genetics, Obstetrics and Gynecology, Herbert Wertheim College of Medicine, Florida International University, Miami, FL 33199, USA; 4 Division of Reproductive Endocrinology, Department of Obstetrics and Gynecology, University of Colorado Anschutz Medical Campus, Aurora, CO 80045, USA

**Keywords:** LH, Leydig cell, LH-receptor, testosterone, spermatogenesis, thrombospondin-2

## Abstract

In the mouse, two distinct populations of Leydig cells arise during testis development. Fetal Leydig cells arise from a stem cell population and produce T required for masculinization. It is debated whether they persist in the adult testis. A second adult Leydig stem cell population gives rise to progenitor-immature-mature adult type Leydig cells that produce T in response to LH to maintain spermatogenesis. In testis of adult null male mice lacking either only LH (*Lhb*^−/−^) or LHR (*Lhr*^−/−^), mature Leydig cells are absent but fetal Leydig cells persist. Thus, it is not clear whether other ligands signal via LHRs in *Lhb* null mice or LH signals via other receptors in the absence of LHR in *Lhr* null mice. Moreover, it is not clear whether truncated LHR isoforms generated from the same *Lhr* gene promoter encode functionally relevant LH receptors. To determine the in vivo roles of LH-LHR signaling pathway in the Leydig cell lineage, we generated double null mutant mice lacking both LH Ligand and all forms of LHR. Phenotypic analysis indicated testis morpho-histological characteristics are identical among double null and single mutants which all showed poorly developed interstitium with a reduction in Leydig cell number and absence of late stage spermatids. Gene expression analyses confirmed that the majority of the T biosynthesis pathway enzyme-encoding mRNAs expressed in Leydig cells were all suppressed. Expression of thrombospondin-2, a fetal Leydig cell marker gene was upregulated in single and double null mutants indicating that fetal Leydig cells originate and develop independent of LH-LHR signaling pathway in vivo. Serum and intratesticular T levels were similarly suppressed in single and double mutants. Consequently, expression of AR-regulated genes in Sertoli and germ cells were similarly affected in single and double mutants without any evidence of any additive effect in the combined absence of both LH and LHR. Our studies unequivocally provide genetic evidence that in the mouse testis, fetal Leydig cells do not require LH-LHR signaling pathway and a one-to-one LH ligand-LHR signaling pathway exists in vivo to regulate adult Leydig cell lineage and spermatogenesis.

## 1. Introduction

Luteinizing hormone (LH) is a pituitary-derived heterodimeric glycoprotein synthesized in gonadotropes. It consists of an α- subunit that is non-covalently associated with the hormone-specific β-subunit [1,2,3]. In the male, LH binds and signals via G-protein coupled receptors (GPCRs) known as LH receptors (LHRs) expressed on Leydig cells within the testis interstitium [1,2,3]. LHR-mediated intracellular signaling cascades regulate a battery of enzymatic steps resulting in testosterone (T) biosynthesis in Leydig cells [1,2,3]. T diffuses into testis tubules and binds androgen receptors (ARs) expressed in Sertoli cells and regulates spermatogenesis and male fertility [4,5].

During mouse testis development, two distinct populations of Leydig cells arise within the testis interstitium [6,7,8,9,10,11,12,13]. The fetal Leydig stem cells give rise to fetal Leydig cells which produce testosterone required for masculinization. Although originally thought these fetal Leydig cells do not persist, the idea that they do exist in the adult testis was confirmed by elegant lineage tracing studies [14,15,16,17]. Subsequently, a distinct adult Leydig stem cell population in the adult testis gives rise to highly proliferative Leydig progenitor cells from which immature Leydig cells are produced. Immature Leydig cells have limited proliferating capacity and terminally differentiate into mature adult Leydig cells. Mature Leydig cells produce testosterone in response to LH to maintain spermatogenesis in the adult [6,7,8,9,12]. The distinct actions of LH- and LHR-mediated signaling in fetal versus adult Leydig cells remain unclear.

Genetically engineered mouse models confirm LH action is essential for spermatogenesis. *Lhb* null mice (and hence LH-deficient) are hypogonadal and infertile [18]. These null mice demonstrate defects in adult Leydig cell development, profound suppression of serum and intratesticular T and spermiogenesis defect [18,19]. However, *Lhb^+/^*^−^ heterozygous mice are fertile and do not show any overt testis phenotypes. Similarly, two different and independent null mutations were engineered at the *Lhr* locus and *Lhr* null mice were generated [20,21]. In both cases, *Lhr* null mutants exhibit hypogonadism and infertility and defects in Leydig cells and steroidogenesis, similar to that observed in *Lhb* null mice [20,21]. *Lhr^+/^*^−^ heterozygous mice are fertile and do not show any overt testis phenotypes, similar to *Lhb^+/^*^−^ heterozygous mice. 

It is interesting to note that in either the LH ligand (*Lhb*) or receptor (*Lhr*) null mice, fetal Leydig cells persist compared to those in control male mice [12,18,20,21]. This raises the possibility that ligands other than LH may signal through LHRs or LH may signal via receptors other than LHR to maintain fetal Leydig cells. Here, we genetically intercrossed heterozygous *Lhb^+/^*^−^ and *Lhr^+/^*^−^ mice and generated *Lhb Lhr* double null mutants. We report testis phenotypes and quantitative expression data on key steroidogenesis enzymes and other testis cell marker genes in these double null mutants. Our studies reveal that a one-to-one LH-LHR signaling exists in vivo in mouse testis and genetically confirm that *Lhb Lhr* double mutant mice do not exhibit any additional testis phenotypes compared to individual null mutants lacking either *Lhb* or *Lhr*. Furthermore, our studies unequivocally confirm that fetal Leydig cells arise independent of LH-LHR-mediated signaling during mouse testis development.

## 2. Results

### 2.1. Generation of Lhb^−/−^ Lhr^−/−^ Double Mutant Mice

In the mouse *Lhb* is localized to chromosome 7 and *Lhr* is localized to chromosome 17. To generate double mutant mice lacking both *Lhb* and *Lhr*, we set up a two-step breeding scheme. We first obtained *Lhb^+/^*^−^ *Lhr^+/^*^−^ double heterozygous mice and subsequently intercrossed these *Lhb^+/^*^−^ *Lhr^+/^*^−^ double heterozygous mice (Supplementary Figure S1). This scheme successfully resulted in generation of *Lhb*^−/−^ *Lhr*^−/−^ double mutant male mice in the expected 1:32 frequency. *Lhb*^−/−^ *Lhr*^−/−^ double mutant male mice were viable and developed normally.

### 2.2. Lhb^−/−^ Lhr^−/−^ Double Mutant Male Mice Display Testis Phenotypes Similar to Single Null Mice Lacking Only Lhb or Lhr

**Figure 1:**
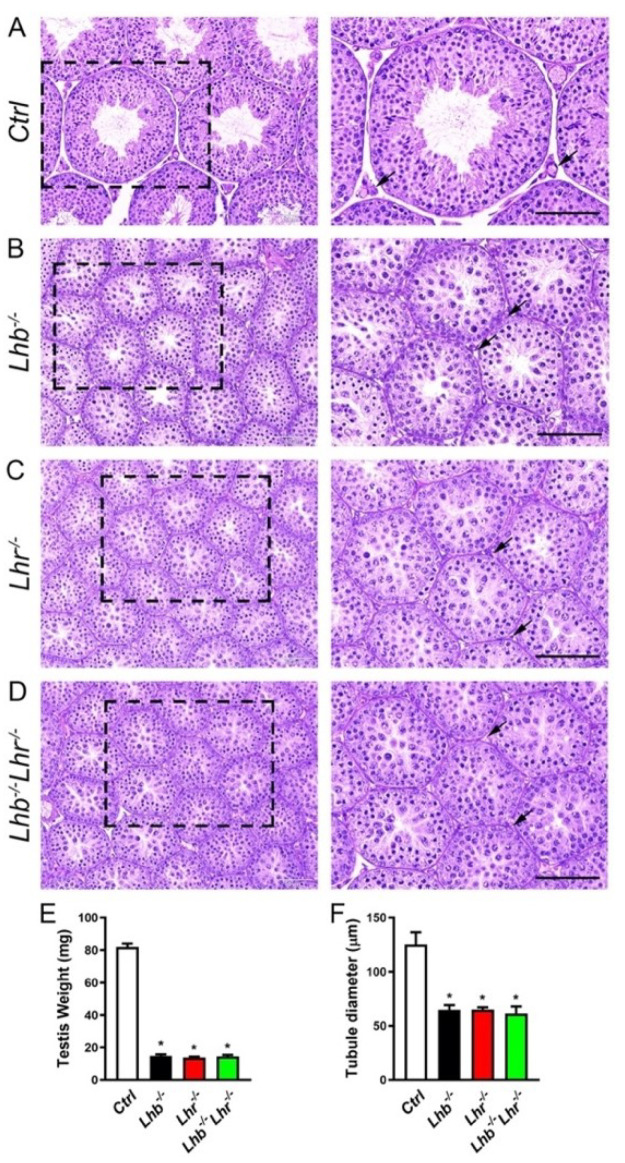
Histo-morphological analysis of testis from single and double null mutant males. PAS-hematoxylin-stained testis sections (**A**–**D**) indicate that compared to the presence of well-defined interstitium (black arrow in high magnification image of panel (**A**)) and all stages of spermatogenesis including late stage spermatids in testis of a control (*Ctrl*) mouse (**A**), the interstitium is poorly developed in single (*Lhb*^−/−^, panel (**B**) and *Lhr*^−/−^, panel (**C**)) and double mutant (*Lhb*^−/−^ *Lhr*^−/−^, panel (**D**)) mouse testis sections (black arrows in high magnification images of panels (**B**–**D**)). Testis weights (**E**) and tubule dimeter (**F**) are significantly reduced in single and double mutant mice compared to controls (* *p* < 0.05, *Ctrl* vs. *Lhb*^−/−^ or *Lhr*^−/−^ or *Lhb*^−/−^ *Lhr*^−/−^, One-way ANOVA) but are similar among single and double mutants (*p* > 0.05, *Lhb*^−/−^ vs. *Lhr*^−/−^ or *Lhb*^−/−^ *Lhr*^−/−^; *Lhr*^−/−^ vs. *Lhb*^−/−^ *Lhr*^−/−^, One-way ANOVA). Testis obtained from adult male mice at 56d of age were used for histo-morphological analysis. Black squares in panels (**A**–**D**) are the regions magnified and represented on the right panels. In panel (**E**): * *p* < 0.05, one-way ANOVA, n = 3. In panel (**F**): * *p* < 0.05, one-way ANOVA, n = 350 tubules from multiple testis sections obtained from 3 mice. Scale bar in panels (**A**–**D**) represents 50 μm.

**Figure 2:**
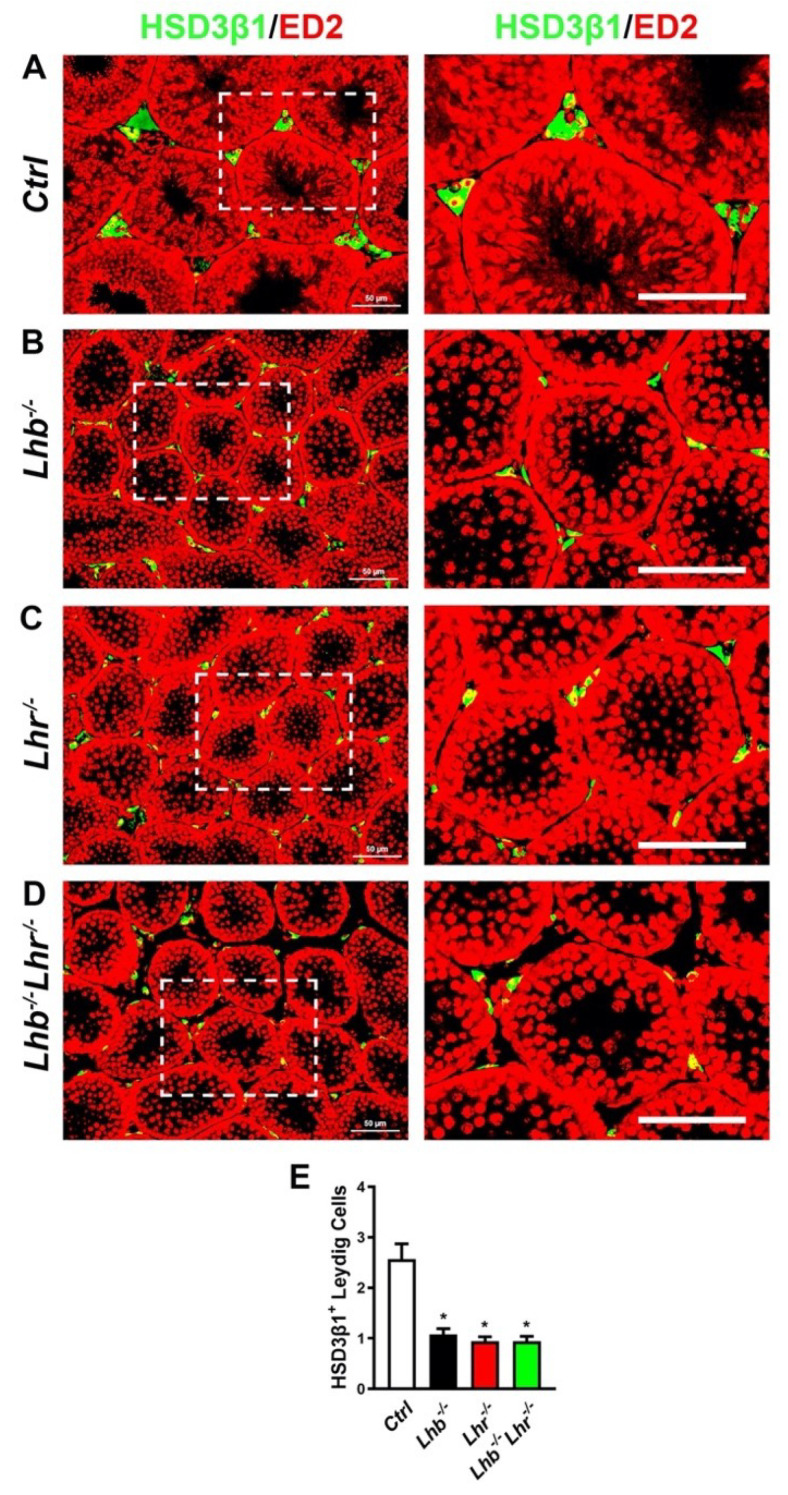
Immunofluorescence analysis of Leydig cells in testis sections. HSD3β1^+^ Leydig cells are visualized in interstitial spaces in testis sections obtained from control mice (*Ctrl*; (**A**)), mice lacking either only LH (**B**), or only LHR (**C**) or both LH and LHR (**D**). Regions indicated in white squares are shown as higher magnification images in right side panels in each case. Nuclei are stained in red. White bar represents 50 μm. HSD3β1^+^ Leydig cells in multiple interstitial spaces are quantified (**E**). Loss of LH or LHR or both results in significant suppression (58–64%) of HSD3β1^+^ Leydig cells compared to the control group (* *p* < 0.05, single or double mutant vs. *Ctrl*; at least 100 interstitial spaces per genotype) but no differences were noted in HSD3β1^+^ Leydig cells between single or double mutant sections (*p* > 0.05, single or double mutant vs. *Ctrl*; at least 100 interstitial spaces per genotype).

Testis histology of single mutant adult male mice lacking either LH ligand (*Lhb*^−/−^*)* or LHR (*Lhr*^−/−^) demonstrate severely reduced interstitium between tubules and spermatogenesis arrest, when compared to testis histology in control WT mice [18,20,21] and as shown in Figure 1. To determine how the combined absence of LH ligand and LHR would affect testis development, we performed histological analysis on testis obtained from *Lhb*^−/−^ *Lhr*^−/−^ double mutant male mice at age 56d and compared to that in age-matched controls (*Ctrl*). Grossly, we did not find any histological differences among PAS-hematoxylin-stained testis sections obtained from *Lhb*^−/−^ or *Lhr*^−/−^ single or *Lhb*^−/−^ *Lhr*^−/−^ double mutants (Figure 1A–C). The interstitium in double mutant testis tubules and lumen were similarly insignificant and spermatogenesis was arrested at the round spermatid stage and elongated spermatids were absent (Figure 1D). Consistent with the testis histology, testis weight (Figure 1E) and tubule diameter (Figure 1F) were significantly suppressed compared to *Ctrl* group (*p* < 0.05, One way ANOVA, *Ctrl* vs. *Lhb*^−/−^ or *Lhr*^−/−^ or *Lhb*^−/−^ *Lhr*^−/−^; n = 3 mice for testis weights and n = 350 tubules for tubule diameter measurements from multiple sections obtained from 3 mice) but were comparable between *Lhb*^−/−^ or *Lhr*^−/−^ single and *Lhb*^−/−^ *Lhr*^−/−^ double mutants (*p* > 0.05, One way ANOVA, *Lhb*^−/−^ vs. *Lhr*^−/−^ or *Lhb*^−/−^ *Lhr*^−/−^; *Lhr*^−/−^ vs. *Lhb*^−/−^ *Lhr*^−/−^. Expression of 3-beta-hydroxysteroid dehydrogenase-1 (HSD3B1) is found in both fetal and adult Leydig cells [22,23,24]. We performed immunofluorescence analysis (Figure 2A–D) on testis sections and counted HSD3B1^+^ Leydig cells within interstitial spaces. There was ~58–64% reduction in HSD3B1^+^ Leydig cells in testis of single or double mutants lacking only LH or only LHR or both LH and LHR compared to control mice (Figure 2E). Thus, testis histo-morphological phenotypes were similar with no additional apparent abnormalities in *Lhb*^−/−^ *Lhr*^−/−^ mice when compared to those in mice lacking only *Lhb* or *Lhr*.

### 2.3. Testicular Gene Expression Changes and T Levels in Lhb^−/−^ Lhr^−/−^ Double Mutant Are Similar to Those in Mice Lacking either Lhb or Lhr

**Figure 3:**
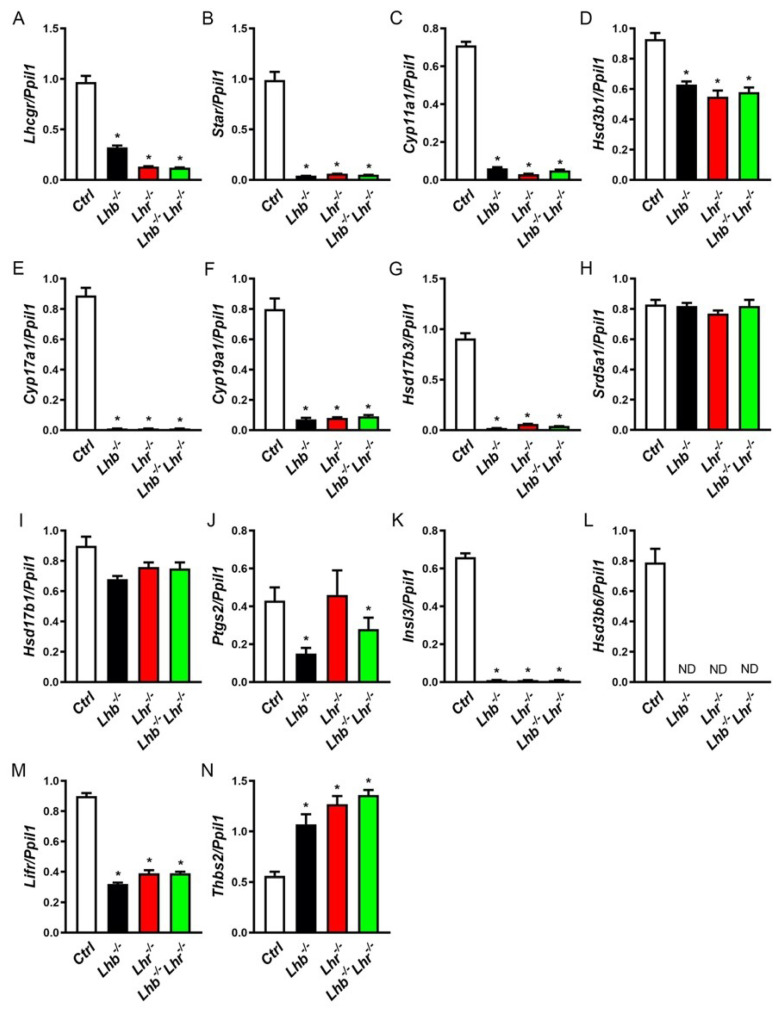
Taqman qPCR analysis of Leydig cell marker genes. Expression of Leydig cell marker genes was quantified in testis samples obtained from adult male mice (n = 3–4) at 56 d of age. Expression of almost all of the selected mature Leydig cell genes was suppressed in the absence of only LH or LHR or both compared to that in *Ctrl* group but is similar among single and double mutants. *Srd5a1* expression was unaffected in the absence of LH-LHR signaling (**H**). Expression of *Hsd17b1* showed a trend towards suppression (**I**). Adult Leydig cell progenitor marker gene *Lifr* was suppressed in single or double mutants compared to that in *Ctrl* (**M**). Expression of *Thbs2* was significantly upregulated in single and double mutants compared to *Ctrl* (**N**) indicating that fetal Leydig cells develop and accumulate in the absence of either only LH or LHR or both. (**A**–**N**) panels: * *p* < 0.05, One-way ANOVA, *Ctrl* vs. *Lhb*^−/−^ or *Lhr*^−/−^ or *Lhb*^−/−^ *Lhr*^−/−^; *p* > 0.05, One-way ANOVA, *Lhb*^−/−^ vs. *Lhr*^−/−^ or *Lhb*^−/−^ *Lhr*^−/−^ ; *Lhr*^−/−^ vs. *Lhb*^−/−^ *Lhr*^−/−^, in all panels except panel J, where a discordant expression was noted. For all qPCR assays, expression of *Ppil1* was used as internal control and cDNA samples in triplicate were used from testis obtained from 3 mice.

Our previous work identified that loss of LH ligand or LHR results in profound changes in gene expression in multiple testis cell types including Leydig, Sertoli and germ cells in male mice [18,20,21,25,26]. To test the effect of combined loss of both the LH ligand and LHR on testis gene expression, we analyzed testis cell type-specific key marker genes by Taqman qPCR analysis (as shown below and Supplementary Figure S2). As predicted, expression of mature adult type Leydig cell marker genes was suppressed in the absence of either LH or LHR in testes of *Lhb* or *Lhr* single null mutants. Loss of both LH and LHR in *Lhb*^−/−^ *Lhr*^−/−^ double mutants did not show any synergistic effect or additional changes in the expression of these marker genes (Figure 3A–G,I–L). One exception noted was that of *Srd5a1* which did not show any change in the absence of LH or LHR or in the combined absence of both LH ligand and receptor (Figure 3H). Similarly, *Hsd17b1* showed a trend towards suppression (Figure 3I). *Lifr* is normally expressed in multiple testis cell types including adult progenitor Leydig cells, Sertoli and peritubular myoid cells [27,28]. Its expression was also similarly suppressed in testis of double mutants as well as single mutants compared to that in controls (Figure 3M). Of the two *Hsd3b* genes, expression of the mature Leydig cell-specific (*Hsd3b6*) but not the fetal onset form (*Hsd3b1*) was profoundly suppressed (Figure 3D,L). Similarly, expression of *Insl3*, an adult mature Leydig cell-specific marker gene was significantly suppressed in both single and double mutants (Figure 3K). 

Most importantly, expression of *Thbs2*, which is the fetal Leydig cell-specific marker was significantly upregulated in testis of single and double mutants compared to that in *Ctrl* mice (Figure 3N). These data indicate that while most of the adult mature Leydig cell marker genes which encode key T biosynthesis enzymes, were similarly suppressed in the absence of either LH or LHR or both, fetal Leydig cell-specific marker gene (*Thbs2*) was upregulated in the absence of LH or LHR or combined absence of both. Thus, fetal Leydig cells accumulate in testis in the absence of LH or LHR or in the combined absence of both LH and LHR.

**Figure 4:**
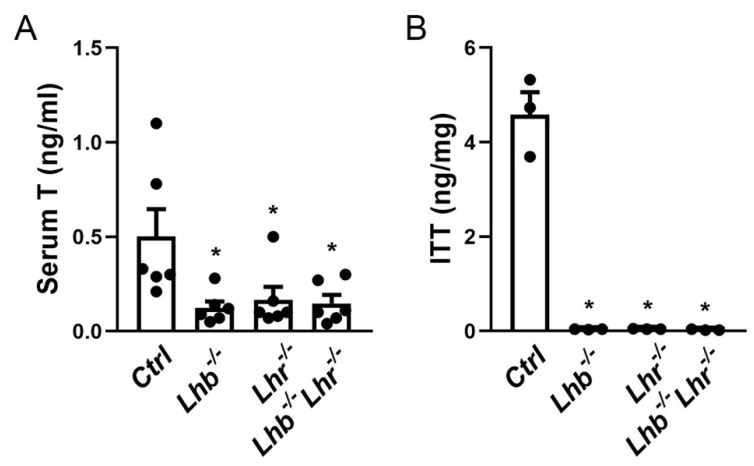
Serum and ITT assays in single and double mutants. Serum (**A**) and intratesticular T (ITT) (**B**) was quantified by an ELISA. Compared to *Ctrl*, both serum (*Ctrl* = 0.5 ± 0.15 ng/mL; *Lhb*^−/−^ = 0.13 ± 0.03 ng/mL; *Lhr*^−/−^ = 0.17 ± 0.07 ng/mL; *Lhb*^−/−^ *Lhr*^−/−^ = 0.15 ± 0.04 ng/mL, * *p* < 0.05, One-way ANOVA, *Ctrl* vs. *Lhb*^−/−^, *Lhr*^−/−^ or *Lhb*^−/−^ *Lhr*^−/−^, n = 4–6 mice) and ITT (*Ctrl* = 4.58 ± 0.04 ng/mg; *Lhb*^−/−^ = 0.04 ± 0.003 ng/mg; *Lhr*^−/−^ = 0.04 ± 0.003 ng/mg; *Lhb*^−/−^ *Lhr*^−/−^ = 0.03 ± 0.006 ng/mg, * *p* < 0.05, One-way ANOVA, *Ctrl* vs. *Lhb*^−/−^, *Lhr*^−/−^ or *Lhb*^−/−^ *Lhr*^−/−^, n = 4–6 mice) are significantly suppressed in single or double mutants. No additive effect of loss both LH and LHR are noted. The serum T and ITT values are not significantly different among single or double mutants (*p* > 0.05, One-way ANOVA, *Lhb*^−/−^ vs. *Lhr*^−/−^ or *Lhb*^−/−^ *Lhr*^−/−^, *Lhr*^−/−^ vs. *Lhb*^−/−^ *Lhr^−/−^,* n = 4–6 mice).

In response to LH stimulation, Leydig cell-derived T diffuses into tubules and binds androgen receptor (AR) in Sertoli cells to regulate spermatogenesis. The clear suppression of most of the T biosynthesis enzyme-encoding mRNAs suggests that T levels must also be suppressed. To further determine, if loss of both LH and LHR results in any additive effect, we quantified both serum (Figure 4A) and intratesticular T (ITT) levels (Figure 4B) by a specific ELISA. Loss of LH or LHR resulted in significant suppression of both serum (Mean ± SEM values are *Ctrl* = 0.5 ± 0.15 ng/mL; *Lhb*^−/−^ = 0.13 ± 0.03 ng/mL; *Lhr*^−/−^ = 0.17 ± 0.07 ng/mL, *p* < 0.05 *Ctrl* vs. single mutant, one-way ANOVA, n = 4–6) and ITT (Mean ± SEM values are *Ctrl* = 4.58 ± 0.04 ng/mg; *Lhb*^−/−^ = 0.04 ± 0.003 ng/mg; *Lhr*^−/−^ = 0.04 ± 0.003 ng/mg, *p* < 0.05 *Ctrl* vs. single mutant, one-way ANOVA, n = 4–6) compared to those in controls as reported earlier [18,20,21], but did not get further suppressed in the combined absence of both LH and LHR in double null mutants (Figure 4A,B). Serum T (0.15 ± 0.04 ng/mL) and ITT (0.03 ± 0.006 ng/mg) values in *Lhb*^−/−^ *Lhr*^−/−^ mice were not significantly different compared to those in single mutants (*p* > 0.05 *Lhb*^−/−^ *Lhr*^−/−^ vs. *Lhb*^−/−^ or *Lhr*^−/−^, one-way ANOVA, n = 4–6). 

**Figure 5:**
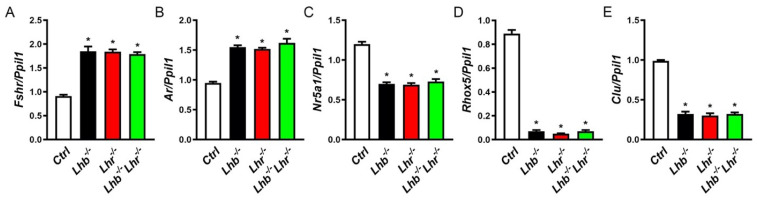
Taqman qPCR analysis of Sertoli cell marker genes. T-repressed genes namely, *Fshr* (**A**) and *Ar* (**B**) are upregulated in the absence of LH or LHR or both. In contrast, those that are positively regulated by T, such as *Nr5a1*, *Rhox5* and *Clu* are similarly suppressed in the absence of LH or LHR or both (**C**–**E**). * *p* < 0.05, One-way ANOVA, *Ctrl* vs. *Lhb*^−/−^ or *Lhr*^−/−^ or *Lhb*^−/−^ *Lhr*^−/−^ ; *p* > 0.05, One-way ANOVA, *Lhb*^−/−^ vs. *Lhr*^−/−^ or *Lhb*^−/−^ *Lhr*^−/−^ ; *Lhr*^−/−^ vs. *Lhb*^−/−^ *Lhr*^−/−^. For all qPCR assays, expression of *Ppil1* was used as internal control and cDNA samples in triplicate were used from testis obtained from 3 mice.

Next, we assessed expression of Sertoli cell-specific marker genes in testis of *Lhb* or *Lhr* single null and *Lhb Lhr* double null mutants. The expression of *Fshr* and *Ar* was under negative regulation of T [29,30,31,32]. Accordingly, testicular expression of *Fshr* and *Ar* was upregulated in the absence of only LH or LHR or combined absence of both LH and LHR (Figure 5A,B). The expression of other androgen-responsive Sertoli cell targets including *Nr5a1*, *Rhox5* and *Clu* was suppressed similarly in single and double null mutants (Figure 5C–E). Thus, absence of LH signaling pathway results in defects in Sertoli cell gene expression secondary to loss of androgen action.

**Figure 6:**
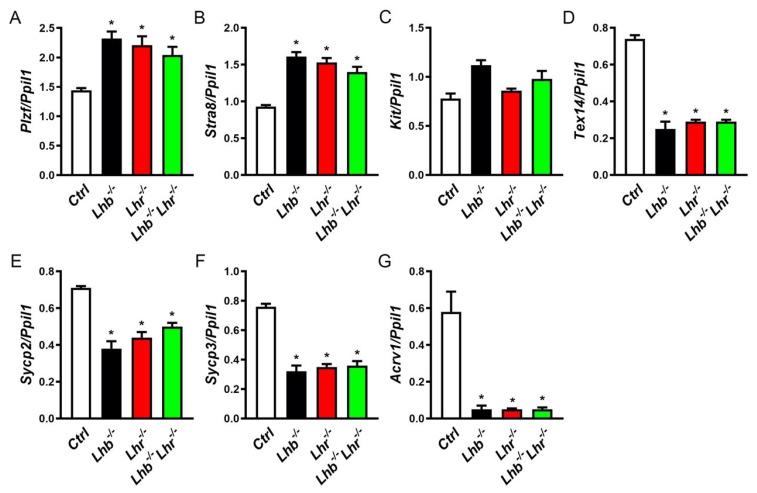
Taqman qPCR analysis of germ cell marker genes. Early germ cell markers such as those expressed in male germline stem cell progenitor (*Plzf*, (**A**)) and spermatocyte (*Stra8*, (**B**); *Kit*, (**C**)) are upregulated or not affected in the absence of LH or LHR or both. Genes that play critical roles in meiosis (*Tex14*, *Sycp2*, *Sycp3*, (**D**–**F**)) and spermatids (*Acrv1*, (**G**)) are significantly suppressed in single and double mutants. No additive effect of loss of both LH and LHR are noted on expression of any of the genes tested. * *p* < 0.05, One-way ANOVA, *Ctrl* vs. *Lhb*^−/−^ or *Lhr*^−/−^ or *Lhb*^−/−^ *Lhr*^−/−^; *p* > 0.05, One-way ANOVA, *Lhb*^−/−^ vs. *Lhr*^−/−^ or *Lhb*^−/−^ *Lhr*^−/−^; *Lhr*^−/−^ vs. *Lhb*^−/−^ *Lhr*^−/−^. For all qPCR assays, expression of *Ppil1* was used as internal control and cDNA samples in triplicate were used from testis obtained from 3 mice.

Finally, we assessed expression of germ cell marker genes by qPCR assays. As predicted from the histological analysis of testis cell types, early stage germ cell markers may not be affected in the absence of LH or LHR or both. Indeed, expression of *Plzf* (stem cell progenitor marker), *Stra8* and *Kit* (spermatocyte markers) was not suppressed in the single or double mutants (Figure 6A–C). Expression of other meiotic / post-meiotic (*Tex14*, *Sycp 2* and *Sycyp 3*) and spermatid (*Acrv1*) markers were significantly suppressed in testis of single and double mutants compared to those in controls (Figure 6D,F–H). Thus, similar to gene expression changes in Leydig and Sertoli cells, combined loss of both LH and LHR did not also result in any additive effects in expression of germ cell marker genes in testis of double mutants.

## 3. Discussion

Earlier studies by us and others established that single mutants lacking genes encoding LH ligand and LHR phenocopy each other [18,20,21]. Using these well-characterized genetic models, here we have generated double mutant mice lacking both the LH Ligand and LHR. This genetic approach allowed us to directly test in vivo LH ligand-independent actions mediated via LHRs and whether LH acts via receptors other than LHR. Our histo-morphological analysis of testis and testicular gene expression analysis in single and double null mutants clearly indicate that combined absence of LH-LHR signaling pathway does not result in any additional phenotypes not observed in single mutants lacking only LH ligand or LHR. Our in vivo genetic approach confirms that it is unlikely LH signals through non-LHRs or other ligands signal through LHR to regulate testis development, spermatogenesis and male fertility. Our *Lhb Lhr* double null mutant mice represent the first genetic model in which signaling via the LH ligand-LH receptor pair is completely absent with only FSH action uniquely present in the male. Additionally, both serum T and ITT are profoundly suppressed in double mutants, similar to those observed in single mutants.

As evident from testicular histology, single and double mutants are indistinguishable. In all three genotypes (*Lhb*^−/−^, *Lhr*^−/−^ and *Lhb*^−/−^ *Lhr*^−/−^), the interstitium is poorly developed with no evidence of adult mature Leydig cells within the interstitium and the lumen is reduced in tubules. Further ultrastructural studies will be needed to confirm the exact morphological identity of the Leydig cell lineage in the absence of only LH or LHR or both. During mouse testis development, two distinct populations of Leydig cells arise. Fetal Leydig cell lineage is present in the absence of only LH or only LHR [18,20,21]. These earlier studies suggested a possibility that non-LH-LHR mediated signaling events may regulate fetal Leydig cells because in single mutants lacking LH ligand, LHRs are present and may mediate actions of ligands other than LH. Similarly, in mutants lacking LHRs, LH ligand is present and may bind receptors other than LHR. The continued presence of fetal Leydig cells as indicated by significantly upregulated *Thbs2* gene expression in double null mutants lacking both LH and LHR and similarly observed in single mutants, provides unequivocal direct genetic evidence that LH-LHR signaling is not required for fetal Leydig cell development in vivo. Our observations on fetal Leydig cells are consistent with those by Shima et al. who used lineage tracing methods and adult *Ar* knockout mice [16] and as summarized [13,24]. It is possible that other ligands such as oxytocin and ACTH and their corresponding signaling pathways may play a role in mouse fetal Leydig cell specification in the absence of LH/LHR [10,11,33,34,35,36,37,38]. Alternatively, other neuroendocrine or locally produced peptides within the testis may also regulate Leydig cells [36,38].

Testis cell type-specific marker gene expression analysis (Leydig cell, Sertoli cell and germ cell-specific markers) revealed identical changes in double null mutants compared to those in single mutants further reinforcing that no additive effects are observed in the combined absence of both LH and LHR. Because we noted both up- and down regulation of genes, these reflect true gene expression changes but not as a result of cell death (Figure 3, Figure 5, Figure 6 and Supplementary Figure S2). Interestingly, expression of *Ptgs2* that encodes prostaglandin synthase-2 is discordant between single and double mutants. Similarly, expression of *Srd5a1* is not altered in single and double mutants compared to that in controls. The exact mechanism for regulating expression of these two enzyme-encoding genes in T biosynthesis pathway remains to be identified. All other mature Leydig cell-, Sertoli- and germ cell- marker genes were identically regulated in the absence of only LH or LHR or both and secondary to absence of T action. Our future RNASeq studies may allow us to identify large-scale gene expression changes and gene networks differentially regulated in testis of single and double mutants.

In summary, genetic analysis of double mutant male mice lacking both LH ligand and LHR reveals identical testis phenotypes observed in individual mutants lacking only LH or only LHR. Significantly upregulated expression of *Thbs2* in testis of single mutants or double mutants lacking both LH and LHR confirms that LH-LHR signaling is not essential for fetal Leydig cell development. Thus, our genetic model provides a novel source of factors that regulate fetal Leydig cells and may provide novel insights into fetal Leydig cell biology and early events in masculinization during testis development. Loss of LH-LHR pathway results in profound suppression of T and pharmacological rescue of single mutants lacking only LH or only LHR by T has already been achieved [18,20,39]. Therefore, our double mutants may prove useful to further understand LH/LHR-independent actions of T in Leydig cell development and spermatogenesis.

## 4. Materials and Methods

### 4.1. Mice

All experimental procedures were carried out on adult male mice (8–10 weeks) and are in accordance with NIH guidelines and approved by the Institutional Animal Care and Use Committee at the University of Colorado Anschutz Medical Campus. *Lhb* null mutant mice were previously generated as described [18]. *Lhr* null mutant mice were generated as previously described [20]. To generate *Lhb Lhr* double null mutants, we initially crossed *Lhb^+/^*^−^ mice with *Lhr^+/^*^−^ mice and obtained *Lhb^+/^*^−^ *Lhr^+/^*^−^ double heterozygous mice. In the second step, we intercrossed these *Lhb^+/^*^−^ *Lhr^+/^*^−^ double heterozygous mice and obtained *Lhb*^−/−^ *Lhr*^−/−^ double mutant mice. Double mutant male mice were used for all present studies and maintained on C57/BL6/129SvEv/129SVJ hybrid genetic background. Mice were housed in rooms equipped with controlled conditions of temperature and humidity and maintained on a 12 h light: dark light cycle with autoclaved standard rodent chow and water supplied ad libitum. For genotyping, tail DNA samples prepared by Millipore (Millipore-Sigma, St. Louis, MO, USA) columns were used in PCR reactions using *Lhb* and *Lhr* allele-specific primer pairs. These primers distinguish the wild-type (WT) and mutant alleles in each case by the size of the amplified DNA fragments separated on ethidium bromide-stained agarose gels as described [20,40].

### 4.2. Histological and Immunofluorescence Analysis

Testes were harvested from adult mice (n = 3) under isoflurane anesthesia, weighed and one testis was immediately fixed in Bouin’s reagent solution (Millipore-Sigma, St. Louis, MO, USA) overnight with constant shaking at room temperature and changed into 70% ethanol. The paraffin-embedded sections were cut at 6 μm thickness, deparaffinized and rehydrated by serial immersion in xylene, followed by graded series of alcohol and stained with periodic acid-Schiff’s reagent (PAS) and hematoxylin as described [18,41,42,43]. The images of stained testis sections were digitally captured using a Leica microscope and used for tubule diameter calculations as described [43,44]. Immunofluorrescence was performed on testes sections using a rabbit antibody (1:2000) against HSD3B1 (gift from Dr. Buck Hales) and counterstained with Ethidium Homodimer-2 (E3599, Invitrogen, Carlsbad, CA, USA) for visualization of nuclei in cells as described [42,45]. A goat anti-rabbit IgG-Alexa flour-488 conjugated second antibody (Invitrogen, Carlsbad, CA, USA) was used at a dilution of 1:200. Antibody-stained sections were mounted with ProLong Diamond Antifade Mountant (P36970, Life Technologies, Carlsbad, CA, USA), and observed under an epifluorescence microscope (Leica). 

### 4.3. RNA Isolation and Taqman qPCR Assays

Total RNA was extracted from testes using RNeasy Mini Kit (74106, QIAGEN, Germantown, MD, USA). One μg of RNA was reverse transcribed into cDNA using SuperScript™—III Reverse Transcriptase (18080-093, Invitrogen, Carlsbad, CA, USA). PCR was performed in 10 μL of reaction volume containing 2 μL of cDNA which was diluted at 1:40, 0.05 μM each of Primer/probe combos, and 5 μL of 2× PrimeTime Gene Expression Master Mix (1055772, Integrated DNA Technologies, Coralville, IA, USA) using QuantStudio 6 Flex Real-Time PCR machine. The relative standard curve method was used for gene expression quantification as described [42,43]. For each primer, a series of dilution of standard cDNA at 1:5, 1:10 and 1:50 were assigned the quantity as 2000, 1000 and 200. Relative mRNA levels of target genes normalized to *Ppil1* expression were obtained and the ratios were presented. Predesigned mouse qPCR Primer/probe combos were purchased from Integrated DNA Technologies, Inc., Coralville, IA, USA. For qPCR assays, triplicate cDNA samples were used from testis obtained from at least 3 mice.

### 4.4. Testosterone Assays 

Mice were exsanguinated under isoflurane anesthesia and serum was separated in a table top centrifuge at room temperature and stored frozen at −80 °C until further use. Frozen testis samples were processed as described [46] and the resulting supernatant aliquots were used for intratesticular T measurements. Total protein in testicular extracts was quantified by BCA protein assay kit (BioRad, Hercules, CA, USA) using bovine serum albumin as standard. Serum (ng/mL) and intratesticular T (ng/mg) levels were measured in 25 μL sample aliquots by an ELISA kit (TE187S-100, Calbiotech, Inc., El Cajon, CA, USA) according to manufacturer’s instructions. Samples were prepared from tissues collected from 3–6 mice. The assay sensitivity is 0.1 ng/mL and intra-assay and inter-assay % CVs are 6.4 and 9.7, respectively.

### 4.5. Statistical Analysis

Data are presented as the mean ± standard error of the mean (SEM). Statistical significance was determined using ANOVA followed by Turkey’s post hoc test. *p* < 0.05 was considered statistically significant. Statistical analyses were performed using PRISM software (version 9.4.1, GraphPad Software, Inc., San Diego, CA, USA).

## Supplementary Materials

The following supporting information can be downloaded at: https://www.mdpi.com/article/10.3390/ijms232415725/s1.

## Abbreviations

AR = Androgen receptor; LH = Luteinizing hormone; LHR = LH-receptor; T = Testosterone
