# Supplementary material for: Lhb−/−Lhr−/− Double Mutant Mice Phenocopy Lhb−/− or Lhr−/− Single Mutants and Display Defects in Leydig Cells and Steroidogenesis
Source: Int J Mol Sci. 2022 Dec 11;23(24):15725. doi: 10.3390/ijms232415725 (PMC9779075; doi:10.3390/ijms232415725)
Supplement: Supplementary file 1 [file ijms-23-15725-s001.zip › ijms-2070587-supplementary.pdf]

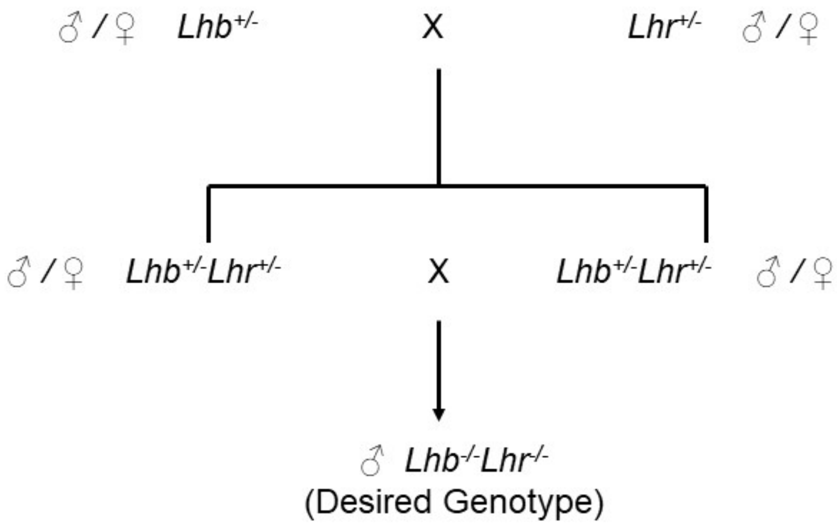

Supplementary Figure S1. A two-step breeding scheme to generate *Lhb* $^{-/-}$ *Lhr* $^{-/-}$ double mutant mice. Initially, *Lhb* $^{+/-}$ and *Lhr* $^{+/-}$ heterozygous mice were bred to generate *Lhb* $^{+/-}$ *Lhr* $^{+/-}$ double heterozygous mice. These mice were then intercrossed to generate the desired *Lhb* $^{-/-}$ *Lhr* $^{-/-}$ double homozygous mutant mice.

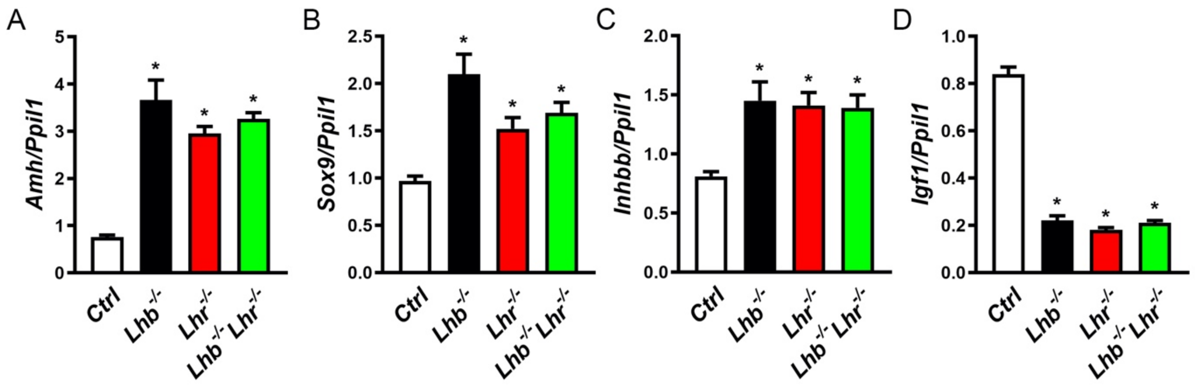

Supplementary Figure S2. Taqman qPCR analysis of additional Sertoli cell marker genes. *Amh* (A) and *Sox9* (B) and *Inhbb* (C) are upregulated in the absence of LH or LHR or both. In contrast, *Igf1* (D) is suppressed in the absence of LH or LHR or both. * $P < 0.05$, One -way ANOVA, *Ctrl* vs. *Lhb* $^{-/-}$ or *Lhr* $^{-/-}$ or *Lhb* $^{-/-}$ *Lhr* $^{-/-}$; $P > 0.05$, One-way ANOVA, *Lhb* $^{-/-}$ vs. *Lhr* $^{-/-}$ or *Lhb* $^{-/-}$ *Lhr* $^{-/-}$; *Lhr* $^{-/-}$ vs. *Lhb* $^{-/-}$ *Lhr* $^{-/-}$. For all qPCR assays, expression of *Ppil1* was used as internal control and cDNA samples in triplicate were used from testis obtained from 3 mice.
